# Supplementary material for: Cardiac Troponin I Antibodies Induce Cardiomyocyte Damage and Alter Cell Morphology
Source: Int J Mol Sci. 2025 Oct 14;26(20):10005. doi: 10.3390/ijms262010005 (PMC12564770; doi:10.3390/ijms262010005)
Supplement: Supplementary file 1 [file ijms-26-10005-s001.zip › Supplement Figures S1-S6 and Table S1.pdf]

Supplementary Materials

Cardiac Troponin I Antibodies Induce Cardiomyocyte Damage and Alter Cell Morphology

Jennifer Furkel [1,2,3,4,5,6,†], Vanessa A. Zirkenbach [1,2,†], Maximilian Knoll [3,4,5,6], Renate Öttl [1], Katrin Rein [3,4,5,6], Amir Abdollahi [3,4,5,6], Norbert Frey [1,2], Mathias H. Konstandin [1,2,*,‡] and Ziya Kaya [1,2,*,‡]

1 Department of Cardiology, Angiology and Pneumology, Heidelberg University Hospital, 69120 Heidelberg, Germany; j.furkel@dkfz-heidelberg.de (J.F.); zirkenbach.vanessa@gmail.com (V.A.Z.); renate.oettl@med.uni-heidelberg.de (R.Ö.); norbert.frey@med.uni-heidelberg.de (N.F.)

2 German Center for Cardiovascular Research (DZHK), Site Heidelberg/Mannheim, 69120 Heidelberg, Germany

3 German Cancer Consortium (DKTK), German Cancer Research Center (DKFZ), 69120 Heidelberg, Germany; maximilian.knoll@med.uni-heidelberg.de (M.K.); katrin.rein@med.uni-heidelberg.de (K.R.); amir.abdollahi@med.uni-heidelberg.de (A.A.)

4 CCU Translational Radiation Oncology, National Center for Tumor Diseases (NCT), Heidelberg University Hospital (UKHD) and German Cancer Research Center (DKFZ), 69120 Heidelberg, Germany

5 Division of Molecular and Translational Radiation Oncology, Department of Radiation Oncology, Heidelberg Faculty of Medicine (MFHD) and Heidelberg University Hospital (UKHD), Heidelberg Ion-Beam Therapy Center (HIT), 69120 Heidelberg, Germany

6 Heidelberg Institute of Radiation Oncology (HIRO), National Center for Radiation Oncology (NCRO), German Cancer Research Center (DKFZ) and Heidelberg University Hospital (UKHD), 69120 Heidelberg, Germany

* Correspondence: mathias.konstandin@med.uni-heidelberg.de (M.H.K.); ziya.kaya@med.uni-heidelberg.de (Z.K.); Tel.: +49-6221-563-5014 (M.H.K.); +49-6221-563-9617 (Z.K.)

† These authors contributed equally to this work.

‡ These authors contributed equally to this work.

Supplemental Figures

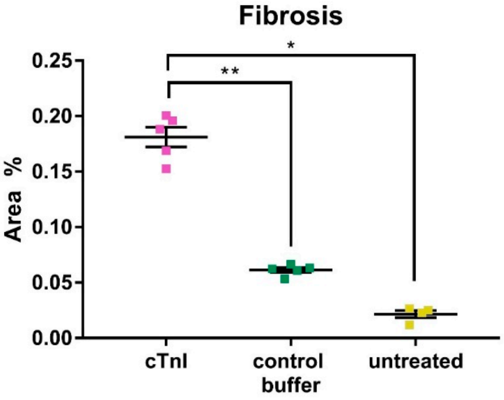

Figure S1. Automated fibrosis area quantification on histopathological sections.

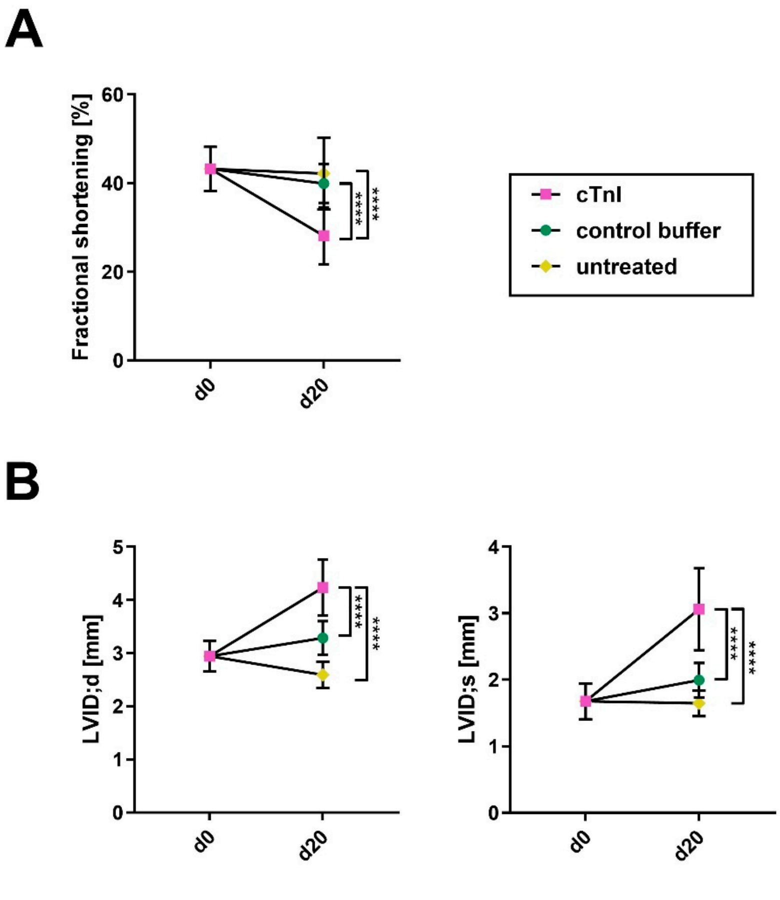

Figure S2. Additional echocardiographical parameters.

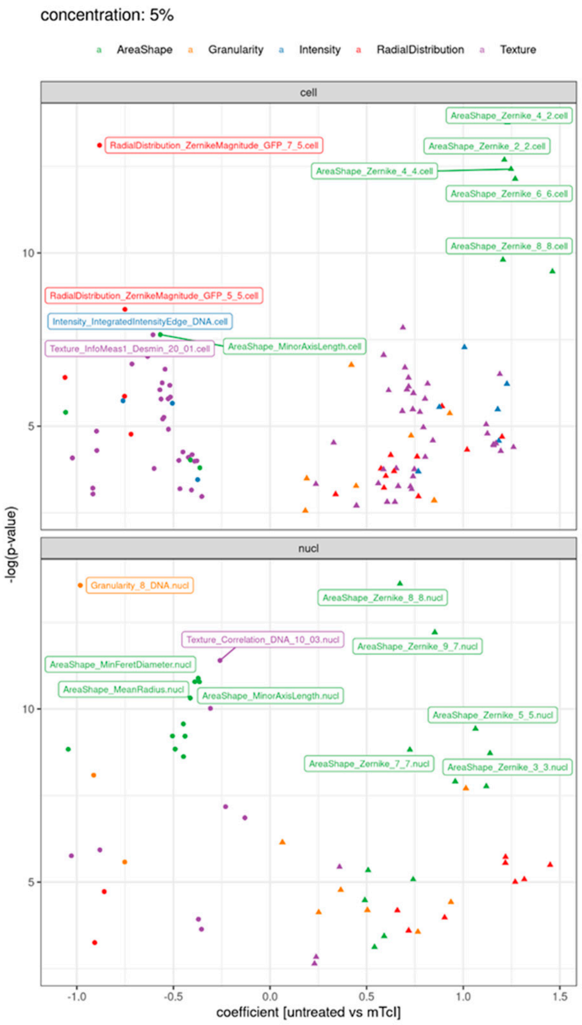
concentration: 5%
AreaShape
Granularity
Intensity
RadialDistribution
Texture
cell
AreaShape_Zernike_4_2.cell
RadialDistribution_ZernikeMagnitude_GFP_7_5.cell
AreaShape_Zernike_2_2.cell
AreaShape_Zernike_4_4.cell
AreaShape_Zernike_6_6.cell
AreaShape_Zernike_8_8.cell
RadialDistribution_ZernikeMagnitude_GFP_5_5.cell
Intensity_IntegratedIntensityEdge_DNA.cell
Texture_InfoMeas1_Desmin_20_01.cell
AreaShape_MinorAxisLength.cell
nucl
Granularity_8_DNA.nucl
AreaShape_Zernike_8_8.nucl
Texture_Correlation_DNA_10_03.nucl
AreaShape_Zernike_9_7.nucl
AreaShape_MinFeretDiameter.nucl
AreaShape_MeanRadius.nucl
AreaShape_MinorAxisLength.nucl
AreaShape_Zernike_5_5.nucl
AreaShape_Zernike_7_7.nucl
AreaShape_Zernike_3_3.nucl
-log(p-value)
10
5
-1.0
-0.5
0.0
0.5
1.0
1.5
coefficient [untreated vs mTcI]

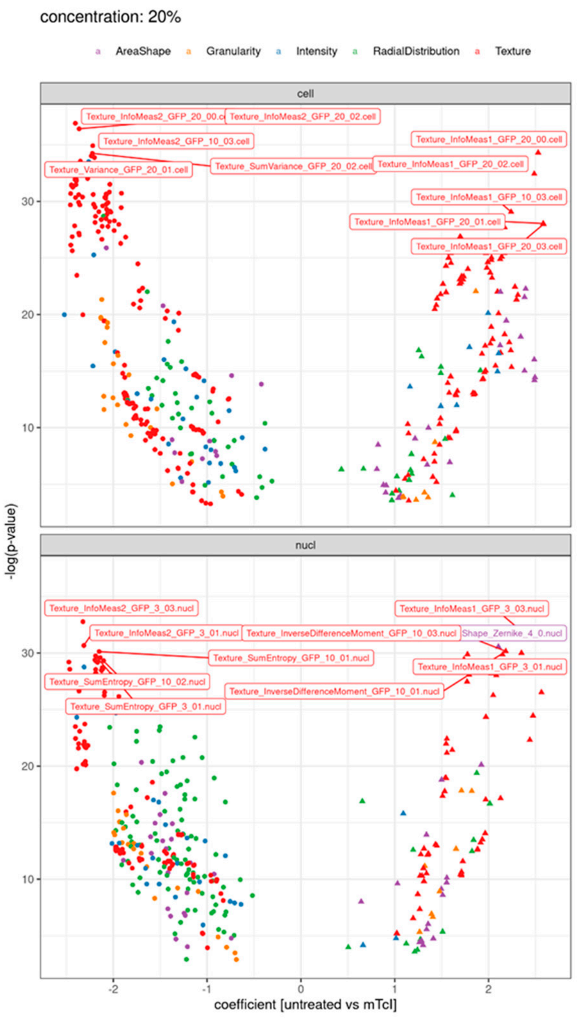

Figure S3. Captured Morphological features. Volcano plots of p-value (log) and effect size of identified differential morphological features for cTnI vs. untreated control NRCMs. Plots are separate for 5 % and 20 % plasma concentration, and for cellular and nuclear features. The color indicates the type of morphological feature: Area and Shape features in green, granularity features in orange, intensity features in blue, Radial distribution features in red, texture features in purple.

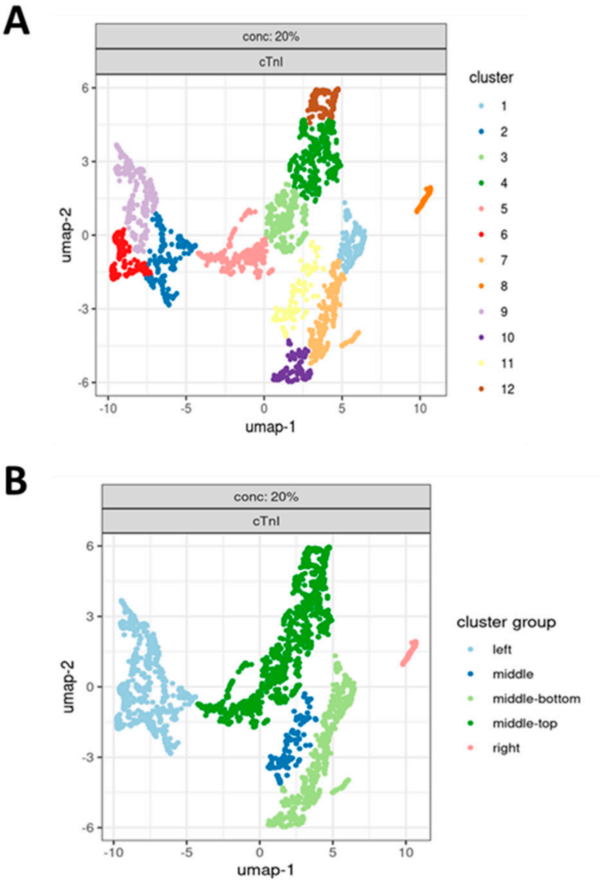

Figure S4. NRCM subtype analysis. Combination of 12 k-means clusters to 5 main clusters showing NRCM subtypes in the cTnI plasma treated NRCMs.

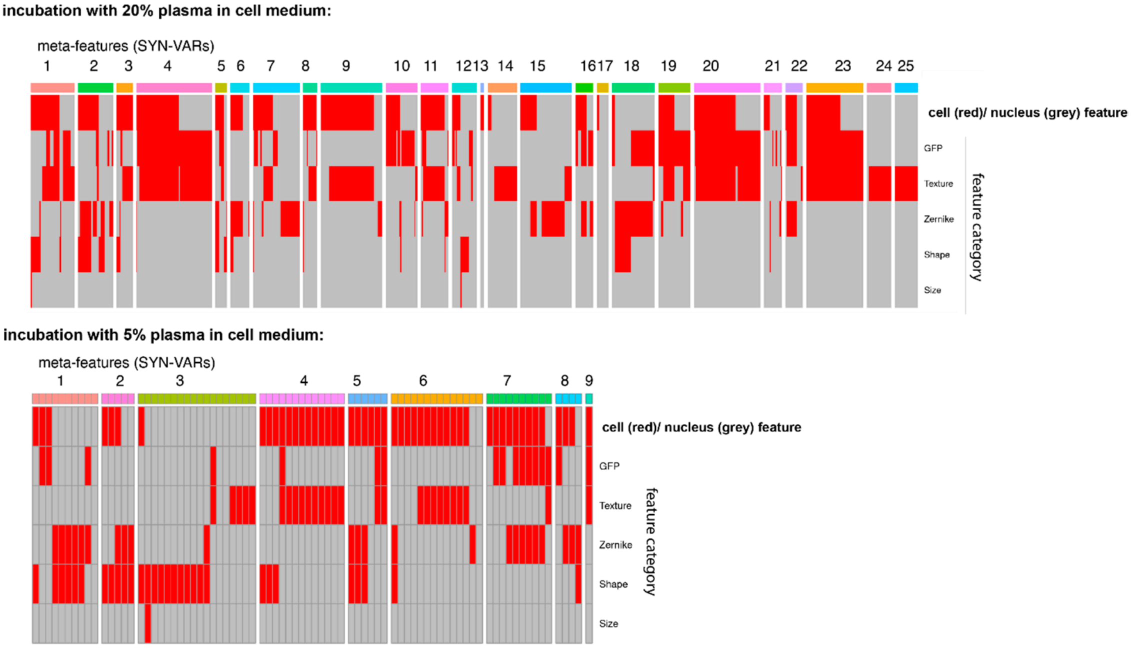

Figure S5. Aggregation of single features into meta-feature (SYN-VARs) to ensure robustness across biological replicates. The y-axis indicates, out of which feature types of single morphological features the meta-features are constructed. The first row indicates in red and grey color, if this single morphological feature is a cellular body (cell, red) or nucleus (grey) measurement. Data is depicted for 5% plasma and for 20% plasma in cell medium. See also Table S2 for feature names.

incubation with 20% plasma in cell medium:

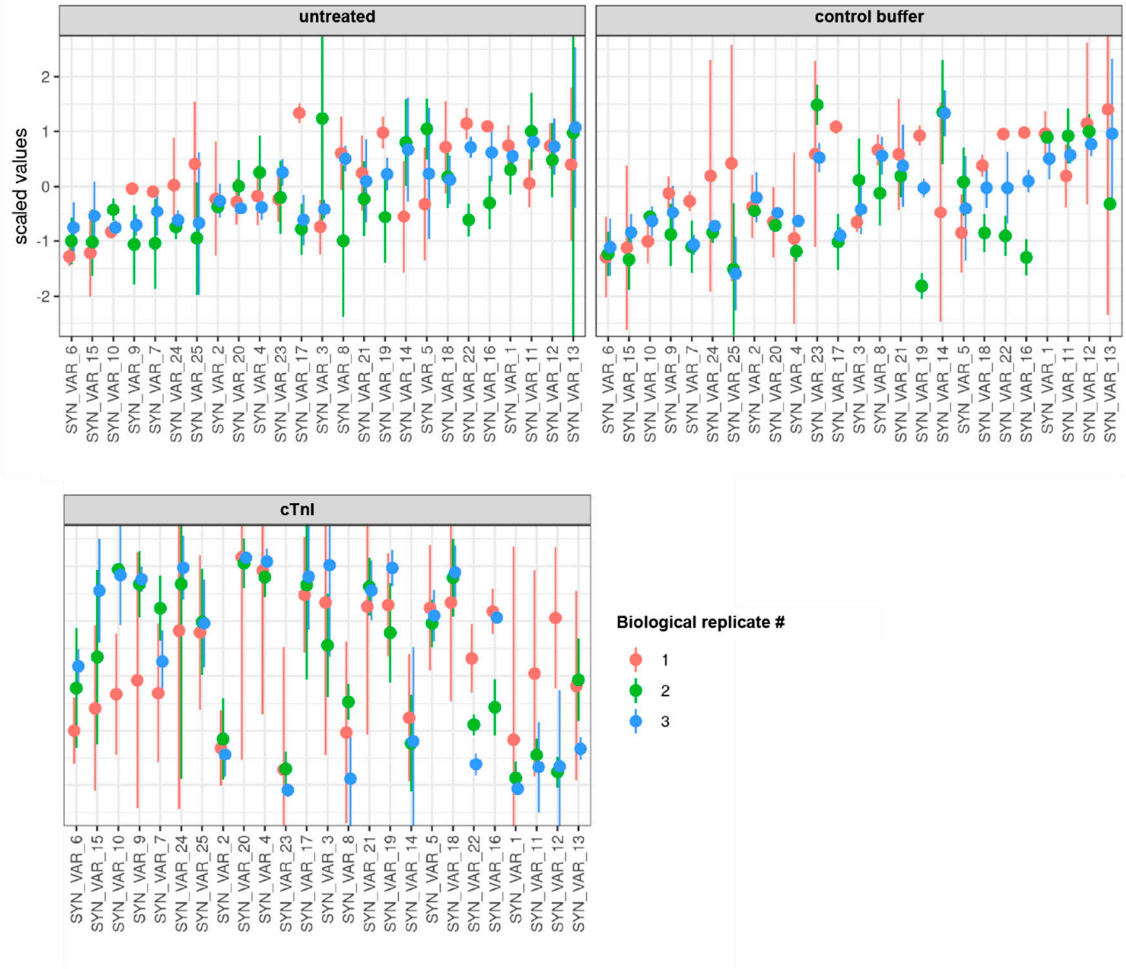

incubation with 5% plasma in cell medium:

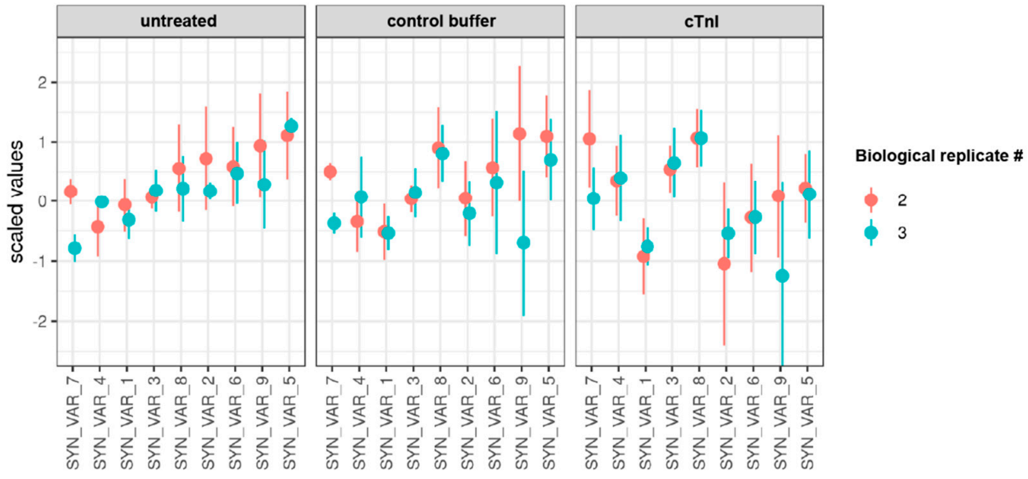

Figure S6. Reproducibility between biological replicates. Meta-features (SYN-VARs/synthetic variables) were calculated from aggregation of single morphological features to ensure robustness. For incubations with 20% plasma we include 3 biological replicates, for incubations with 5% plasma we include 2 biological replicates. The figures shows meta-feature mean values and 95% confidence invervals (CI) for each biological replicate for all three treatment conditions (untreated, control buffer, cTnI). Meta-features are shown ordered by mean values of the untreated controls group.

Supplemental Tables

Table S1. Top 10 cell features. Significantly altered cell features in NRMCs after incubation with cTnI treated mouse plasma for 48 h.

**Features**
Area shape_maximum radius cells
Area shape_minor axis length cell
Area shape Zernike 3_3 nucl
Area shape Zernike 5_5 nucl
Granularity
Radia Distribution Zernike Magnitude GFP 1_1 cell
Area shape Zernike 9_9 nucl
Area shape mean radius cell
Area shape zernike 9_1 nucl
